# 610 Myofibroblasts Are Not Characteristic Features of Keloid Lesions

**DOI:** 10.1093/jbcr/irac012.238

**Published:** 2022-03-23

**Authors:** Dorothy M Supp, Jennifer M Hahn, Kevin L McFarland, Kelly A Combs, Heather Powell

**Affiliations:** University of Cincinnati College of Medicine, Cincinnati, Ohio; University of Cincinnati College of Medicine, Cincinnati, Ohio; University of Cincinnati College of Medicine, Cincinnati, Ohio; University of Cincinnati College of Medicine, Cincinnati, Ohio; The Ohio State University, Columbus, Ohio

## Abstract

**Introduction:**

Keloids are disfiguring, fibroproliferative lesions that can result from an abnormal wound healing process. Keloids are characterized by excessive and abnormal deposition of extracellular matrix and unrestrained expansion beyond the original wound boundary. Keloids are challenging to treat, with low response rates to current interventions and high recurrence rates. Development of more effective, targeted therapies will require a deeper understanding of the mechanisms driving keloid pathology. It has been widely reported that keloids are characterized by the presence of myofibroblasts, which are specialized, contractile fibroblasts that express alpha-smooth muscle actin (αSMA) and play important roles during wound healing and tissue remodeling. However, the evidence supporting a role for myofibroblasts in keloid development is inconclusive, with conflicting reports in the literature. This complicates development of more effective therapies, as the benefit of interventions targeting myofibroblasts is unclear. The current study evaluated the presence of αSMA+ myofibroblasts in keloids, and analyzed αSMA expression in keloid-derived fibroblasts, to determine whether myofibroblast presence can be considered diagnostic of keloid lesions.

**Methods:**

Keloid, hypertrophic scar (HTS), and normal human skin samples were collected with Institutional Review Board approval and primary fibroblast cultures were established. Myofibroblasts in tissue sections were localized by αSMA immunostaining, and αSMA expression in pericytes of blood vessels was distinguished from αSMA+ myofibroblasts by collagen IV immunostaining of vessels. Expression of αSMA mRNA (ACTA2 gene) was measured using quantitative real-time PCR. Statistical analysis via t test was performed using SigmaPlot.

**Results:**

Normal human skin samples did not exhibit αSMA+ myofibroblasts (0/8), but myofibroblasts were identified in 53% of keloids (8/15) and 60% of HTS (3/5) tissue samples. When present in keloids or HTS, the αSMA+ cells were not uniformly distributed, but were often observed in clusters in the deeper regions of the lesions. Comparison of ACTA2 mRNA expression did not reveal any significant differences between normal skin and keloid tissues (N=3/group), or between normal fibroblasts (N=7 donors) and keloid fibroblasts (N=9 donors).

**Conclusions:**

The results suggest that although many keloids contain αSMA+ myofibroblasts, these cells do not appear to be diagnostic for keloids. Therefore, therapies that target myofibroblasts may not be effective for all keloid lesions. However, a limitation of this study is that only excised keloid and HTS tissues, which tend to be larger, more mature lesions, were examined; thus we cannot rule out a potential role for myofibroblasts in early development of abnormal scars.

